# Practical insight into upright breech birth from birth videos: A structured analysis

**DOI:** 10.1111/birt.12480

**Published:** 2020-01-20

**Authors:** Anke Reitter, Alexandra Halliday, Shawn Walker

**Affiliations:** ^1^ Obstetric and Prenatal Medicine Department Hospital Sachsenhausen Academic Teaching Hospital of the Goethe‐University Frankfurt Frankfurt Germany; ^2^ Florence Nightingale Faculty of Nursing, Midwifery and Palliative Care King’s College London London UK; ^3^ Women’s and Children’s Services St Thomas’ Hospital London UK

**Keywords:** algorithm, birth videos, breech presentation, intrapartum care, mechanisms

## Abstract

**Background:**

We aimed to identify common features of upright vaginal breech births with good outcomes to refine a physiological approach to teaching breech birth.

**Methods:**

We performed a structured analysis of 42 videos of successful upright breech births (eg, kneeling, hand/knees), facilitated by obstetricians (n = 34) and midwives (n = 8) in nine different countries. Precise timings and relevant clinical details were recorded on an Excel spreadsheet. Each video was analyzed twice by at least two members of the research team. Time‐to‐event intervals, frequencies of interventions, and descriptive statistics were calculated using SPSS.

**Results:**

A completely spontaneous (labor mechanisms and maternal effort only) birth occurred in 14/42 (33%) cases. The median time between the birth of the fetal pelvis and the head in all births was 1:52 (IQR 1:05,2:46; min:sec). Lack of spontaneous rotation to a sacro‐anterior position by the time the fetus had emerged to the nipple line was strongly associated with fetal arm entrapment. The following maneuvers were used: shoulder press to flex the aftercoming head in midpelvis or outlet (n = 24), sweeping down arm/s (n = 12), buttock lift to assist shoulder press (n = 6), modified Mauriceau (n = 6), rotational maneuvers to release an entrapped arm (n = 6), elevate and rotate fetal head to assist engagement (n = 2), and conversion into supine maternal position (n = 2).

**Conclusions:**

Most upright breech births occur within 3 minutes of the birth of the fetal pelvis. Upright breech birth attendants use variations of traditional maneuvers. We introduce a physiological breech algorithm as an initial timekeeping framework for teaching, research, and practice.

## 1 INTRODUCTION

Whereas the increased perinatal morbidity and mortality from birth trauma and neonatal hypoxia associated with vaginal breech births are well known,^1^ guidance on how and when to intervene has traditionally been based on the opinions of experienced professionals.^2^, ^3^, ^4^, ^5^ However, observation from personal experience is often based on small samples and prone to recall and confirmation bias.^6^ Reviews of neonatal mortalities associated with term vaginal breech births often identify suboptimal care, and delay is often implicated.^7^, ^8^ Identification of common characteristics of births with good outcomes may provide additional insight.

The increasing use of birth videos for training^9^ offers opportunities to identify optimal progress for vaginal breech births through detailed analysis of birth timings and mechanisms. Upright birthing positions for vaginal breech births have been advocated by some obstetricians and midwives, in part due to the clear view of birth progress and fetal condition.^10^, ^11^, ^12^, ^13^ Upright births are also assisted by the effects of gravity and changes in diameters of the maternal pelvis that occur with maternal movement.^14^ Recognizing the characteristics of “normal” breech birth physiology may enable birth attendants to avoid unnecessary and potentially traumatic manipulation. Clearly recognizing when progress deviates from “normal” may also encourage attendants to apply timely assistance when required.

We aimed to perform a structured analysis of birth videos to identify common characteristics among successful breech births, in order to refine a physiological approach to teaching breech birth.^11^

## 2 METHODS

Our sample included 42 videos where women birthed breech‐presenting infants in upright positions (eg, kneeling, hands/knees). Of these, 37/42 (88%) videos were contained in our personal teaching collections, used with consent, anonymized, and in semi‐public circulation on the Breech Birth Network Vimeo teaching platform accessible to trained health care professionals via password. They were donated for use in research in teaching either by practitioners, who obtained local consent, or by women themselves, who intended them to contribute to understanding and increased safety of vaginal breech birth. Others were publicly available online on other platforms (5/42; 12%).

Although we were not provided with specific outcome data, we were informed that all births had good outcomes. We included all birth videos of upright breech births that we were able to locate during the study period (May 2017‐July 2019), had permission to use, and showed the birth continuously from pelvis to head. We attended fewer than half of the births ourselves (AR n = 18; SW n = 2). The videos originated in Germany (22), USA (6), UK (4), Brazil (3), Belgium (3), New Zealand (1), Israel (1), Ecuador (1), and Spain (1). Because the videos were already completely anonymized and in circulation, this study qualified as an analysis of pre‐existing data, not subject to review by a Research Ethics Committee.

Our research team included a Lead Obstetrician in a hospital in which vaginal breech births account for >6% of the total birth rate because of late‐term transfers of care (AR); a Consultant Breech Specialist Midwife in a large teaching hospital (SW); and a Research Assistant (AH) who was trained in physiological breech birth methods and video analysis. Each video was initially analyzed by AR, SW, or AH and was checked for accuracy by SW or AR. A data collection tool was created using Microsoft Excel spreadsheet software. This database comprised more than 80 items, including the time intervals between the birth of the fetal pelvis, umbilicus, arms, and head, as well as any interventions performed during the birth. After an initial analysis, the team met to discuss and modify the data collection tool. A second analysis of each video and accuracy check was completed. Time‐to‐event intervals, frequencies, and descriptive statistics were calculated using IBM SPSS Statistics software (Version 23). Fisher exact test was used to determine association between variations in fetal mechanisms and performance of maneuvers.

In this study, a spontaneous birth is defined as one resulting from labor mechanisms and maternal effort alone, in which no direct manipulation of the fetus occurred.

## 3 RESULTS

### 3.1 Timings

In our sample, the median length of time between the birth of the fetal pelvis and the head was 1:52 (min:sec; IQR 1:05‐2:46) (Table 1). Among spontaneous births only, it was 1:02 (IQR 0:23‐1:31). The median length of time between the birth of the umbilicus and the head was 1:26 (IQR 0:45‐2:17) and 0:39 (IQR 0:13‐1:31) among spontaneous births only. Data were not normally distributed, so the median and intraquartile ranges are reported as the measures of central tendency (Figure S1).

**Table 1:** Timings: Physiological breech birth intervals

Time between	N	Median	Minimum	Maximum	Quartiles	
	Valid				25	75
Birth of fetal pelvis to birth of head	36	01:52	00:06	07:37	01:05	02:46
Birth of fetal pelvis and birth of head in spontaneous births	11	01:02	00:06	02:36	00:23	01:31
Birth of umbilicus to birth of head	42	01:26	00:04	06:53	00:45	02:17
Birth of umbilicus to birth of head in spontaneous births	14	00:39	00:04	05:13	00:13	01:31
Buttocks not receding (“rumping”) to birth of head	21	02:20	01:17	08:06	01:48	04:42
Buttocks not receding to birth of head in spontaneous births	6	01:58	01:25	03:32	01:37	02:37
First and second legs	35	00:00	00:00	00:49	00:00	00:07
First and second legs in spontaneous births	12	00:00	00:00	00:34	00:00	00:03
First and second arms	42	00:02	00:00	02:26	00:01	00:03
First and second arms in spontaneous births	14	00:01	00:00	00:22	00:00	00:05
Birth of fetal pelvis to birth of umbilicus	36	00:18	00:02	03:09	00:07	00:47
Birth of fetal pelvis to birth of umbilicus in spontaneous births	12	00:07	00:02	01:37	00:06	00:31
Buttocks visible to not receding between contractions (“rumping”)	9	01:44	00:00	10:32	00:19	03:26
Buttocks visible to not receding in spontaneous births	2	00:31	00:26	00:36	n/a	n/a
Buttocks not receding to birth of fetal pelvis	21	00:38	00:00	04:25	00:14	01:50
Buttocks not receding to birth of fetal pelvis in spontaneous births	7	00:38	00:04	01:58	00:20	01:38

### 3.2 Fetal mechanisms

In the videos, we were able to observe the following mechanisms, each of which occurred in over half of the sample:

- Birth of the fetal pelvis with the sacrum transverse
- Sacro‐anterior rotation of the fetal pelvis with descent
- Extended legs born simultaneously by hip extension
- Full body recoil flexion: From an extended position, the fetus flexes his/her hips/legs, adducts his/her arms, and flexes his/her head, recoiling to the previous fetal position
- Flexed arms born across the body with the shoulders in the transverse diameter
- Head born by further flexion and maternal effort

**Figure 1:**
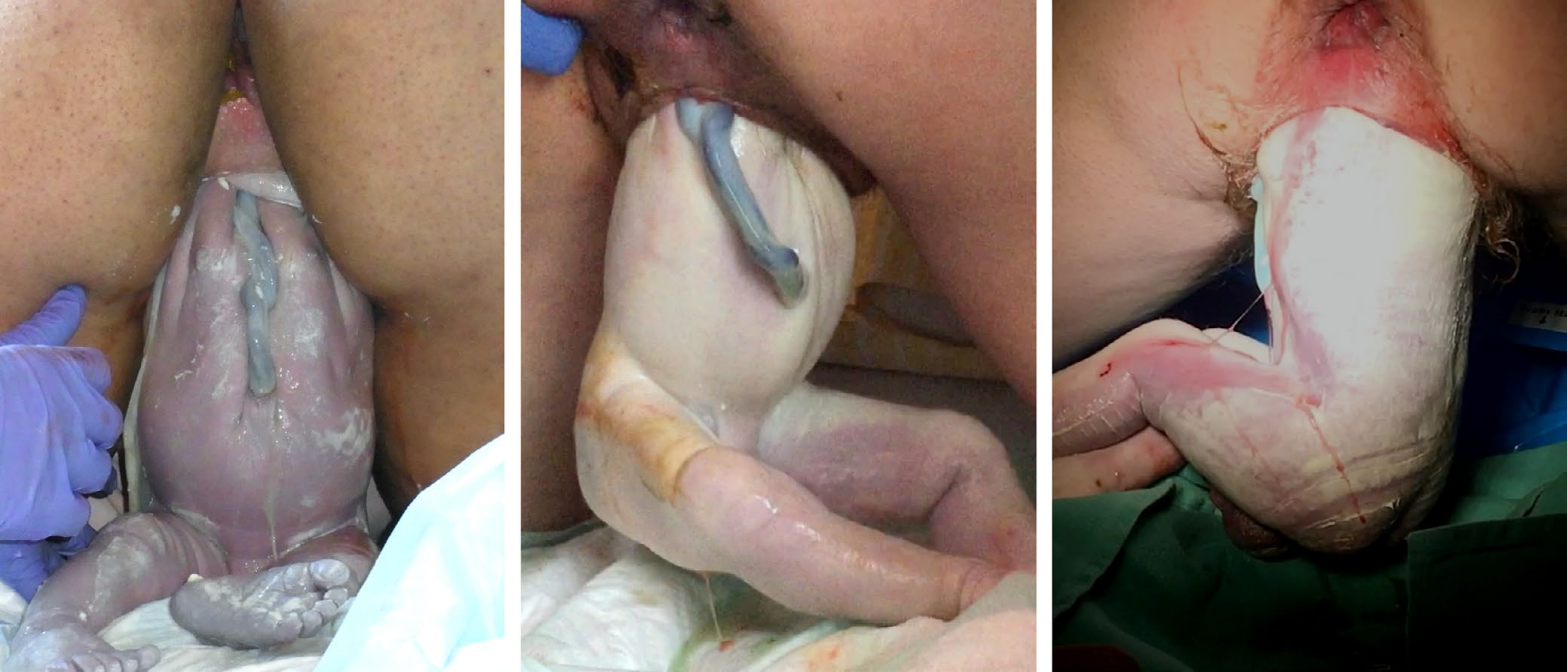
Variations of sacral rotation at the nipple line. From left to right: (1) complete sacro‐anterior rotation; (2) partial sacro‐anterior rotation into oblique; (3) no rotation, sacro‐transverse. *Image credits: Shawn Walker, Anke Reitter, Emiliano Chavira*

In our sample, the fetal sacrum most often emerged in a transverse position (27/42; 64%), followed by oblique (15/42; 36%). In no cases did the sacrum emerge in a direct sacro‐anterior or sacro‐posterior position. After emergence of the fetal pelvis, in 28/42 (67%) cases the fetus began to rotate in a sacro‐anterior direction with further descent. At the time of the birth of the umbilicus, only 7/42 (17%) cases had rotated completely into a direct sacro‐anterior position. By the time the fetus had emerged to the nipple line, 27/42 (64%) had rotated completely spontaneously to sacro‐anterior, and only 3/42 (7%) remained sacro‐transverse (Figure 1).

Among the cases where spontaneous rotation to sacro‐anterior had completed by the nipple line, in only 4/27 (15%) were maneuvers performed to release one or both of the arms, compared with 10/15 (75%) of cases where it had not. In all three cases of no rotation after birth of the pelvis (persistent sacro‐transverse), rotational maneuvers were performed to release entrapped fetal arms. Lack of spontaneous rotation to sacro‐anterior position by the nipple line was associated with use of manual maneuvers to release fetal arms (*P* = .0015).

The fetal back began on the maternal right in 23/42 (55%) cases and on the maternal left in 19/42 (45%) cases. Maneuvers were used at a similar frequency for both positions (right side 16/23, 70%; left side 14/19, 74%; *P* = 1).

### 3.3 Maneuvers

In our sample of upright breech births, a spontaneous birth, defined as no direct maneuvers used, occurred in 14/42 (33%) cases overall, including 10/34 (29%) cases attended by obstetricians and 4/8 (50%) cases attended by midwives (Table 2). We differentiated direct maneuvers, which involved manipulating the baby, and indirect maneuvers, which involved manipulating the woman (Table S1). Indirect maneuvers were only performed by obstetricians and occurred in 15/34 (44%) of obstetrically managed births. In 11/15 (73%) of these cases, further direct maneuvers were also performed.

**Table 2:** Maneuvers and interventions performed in upright physiological breech births

Maneuver/Intervention	Incidence/42	Percentage
Shoulder press—pressure just below the fetal clavicle to move the shoulder girdle back between the mother's legs, to flex the aftercoming head in midpelvis or outlet	24	57
Sweeping down fetal arm/s	12	26
Manually “stretching” the maternal perineum	11	26
Fundal pressure—downward pressure on the maternal abdomen	10	24
Buttock lift to assist shoulder press—lifting maternal buttocks up toward the sacrum, sweeping the perineum over the fetal forehead	6	14
Modified Mauriceau‐Smellie‐Veit/Mauriceau‐Cronk—manually flexing the fetal head by elevating the occiput and downward pressure on the maxilla	6	14
Rotational maneuvers to release an entrapped fetal arm	6	14
Elevate and rotate fetal head to assist engagement in the maternal pelvis—elevating the fetal head at the occiput to raise it off the pelvic inlet and/or internal manual rotation of the occiput to oblique/transverse to assist the head to engage, then rotating the head back to the OA diameter to realign in the midpelvis to deliver the fetal head	2	5
Conversion into supine maternal position	2	5
Handing over to a more experienced professional	2	5
Scoop and flex—internal flexion of the fetal head by sweeping one hand over the parietal bone and pressing down on the forehead (sinciput)	1	2
Episiotomy	1	2

We were able to record the timings of birth of both legs in 35/42 (83%) cases (Table 1). In our sample, the median time between the birth of one leg and the other was 0 second, as over half of our videos showed the legs being born simultaneously. In seven cases, clinicians lifted the perineum to create space for the legs to be born spontaneously. The indication for this was unclear, but in each of these cases (7/7; 100%) further interventions were performed, including assisting the arms in five cases and the head only in the other 2.

Both fetal arms were also born at approximately the same time in completely spontaneous births (Table 1). The median time difference between the birth of the first arm and the second was 2 seconds. In 8/42 (19%) cases, neither arm delivered spontaneously, and in 7/8 (88%) of these cases, clinicians attempted delivery of the sacral arm first. However, in 3/7 (43%) of cases where release of the sacral arm was attempted first, the attempt was abandoned unsuccessfully after 13, 31, and 40 seconds. Switching strategy to release the pubic arm first resulted in both arms being released within 5, 20, and 4 seconds, respectively.

In all remaining (4/7;57%) cases where sweeping release of the sacral arm was successful, further rotational maneuvers were required to release the pubic arm. In these cases, the total time required to release both arms was 30, 40, 65, and 66 seconds. In one case, release of the pubic arm through rotation was performed before attempting to sweep down the sacral arm; in this case, the birth was complete within 33 seconds of initiating the rotational maneuver. When rotational maneuvers were used, they were used to release the pubic arm in all 6/6 (100%) cases.

Full fetal body rotational maneuvers were performed using “flat hands” (1/6—one flat hand on the anterior aspect of the fetal torso, and one on the back, with finger pads along the clavicle and shoulder blades) or “shoulder girdle grip” (5/6—the fetal shoulder girdle is gripped with thumbs anterior and fingers wrapped around the shoulder blades). Rotations were performed by rotating the fetal body either 180° through sacro‐anterior (fetal sacrum to maternal pubis) and 90° back, as described by Louwen (3/6),^15^ or 90° sacro‐posterior (fetal sacrum to maternal sacrum) and 180° back (3/6), as described by Reitter and Walker.^16^, ^17^ After both maneuvers, the fetal body and head were realigned to an occipito‐anterior position for delivery of the head.

Among the 24 instances (57%) shoulder press was used, the median time required to perform the maneuver and deliver the fetal head was 9 seconds (IQR 0:03, 0:15). Either a “fingers below the clavicle” (10/24; 42%) or the “thumbs below the clavicle with fingers wrapped around the shoulder girdle” (14/24; 58%) methods were used.^9^ In 8/24 (33%) cases, the clinician alternated between steady and relieved pressure, “rocking” the fetal head into flexion. A variation of the Mauriceau maneuver to flex the head was performed in 6/42 (14%) cases; this has been referred to as Mauriceau‐Cronk when performed with the woman in an upright position.^18^ In 5/24 (21%) cases, the clinician placed a finger in the mouth of the fetus while performing a head flexion procedure at the pelvic outlet (shoulder press or modified Mauriceau).

Mothers were asked to turn into a supine position on two occasions (2/42; 5%), one before delivery of the arms and one before the delivery of the head. The births were completed 50 and 20 seconds after initiating the position change, respectively.

### 3.4 Algorithm

**Figure 2:**
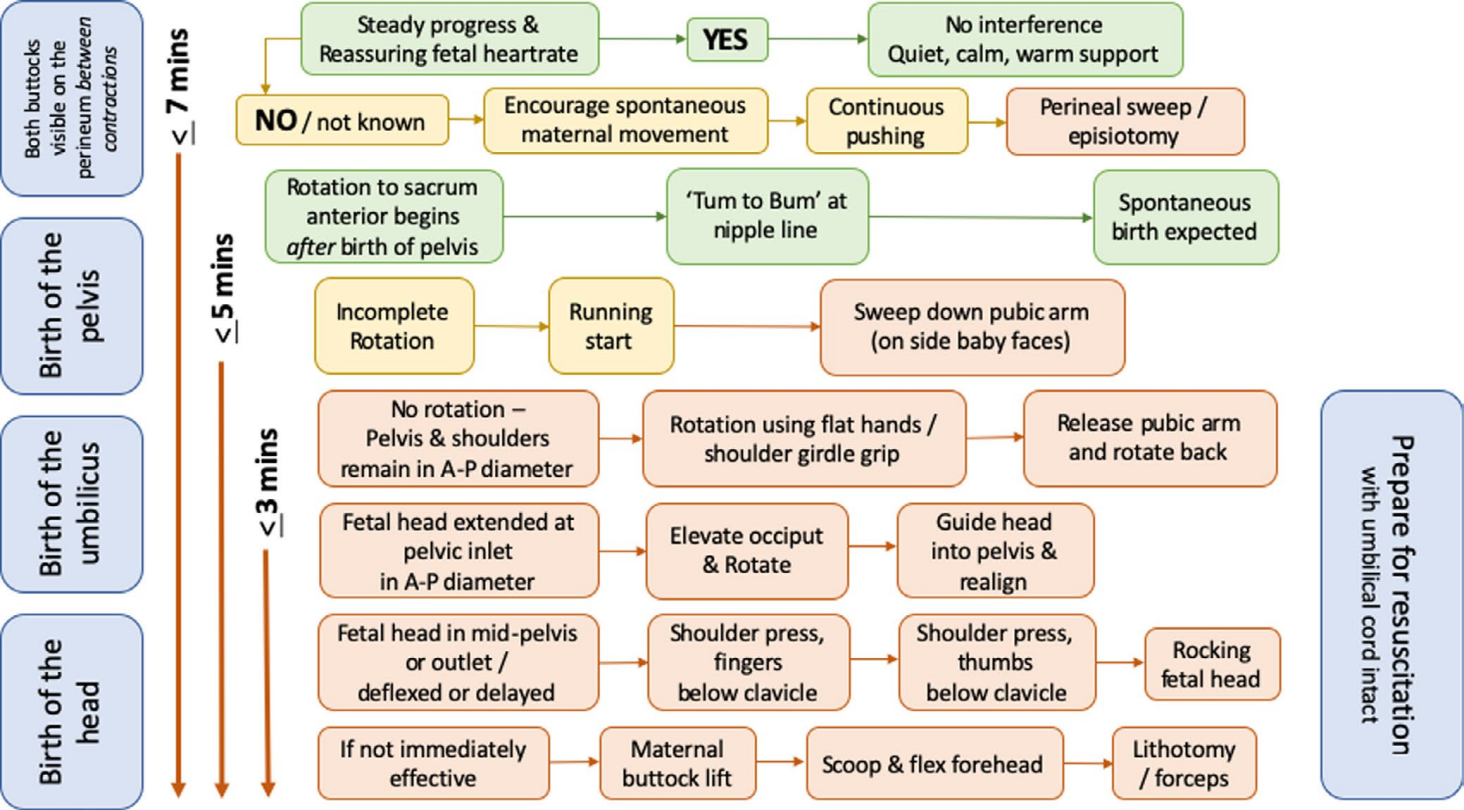
Physiological Breech Birth Algorithm. Designed by Shawn Walker, RM PhD, version: Nov 2019

**Figure 3:**
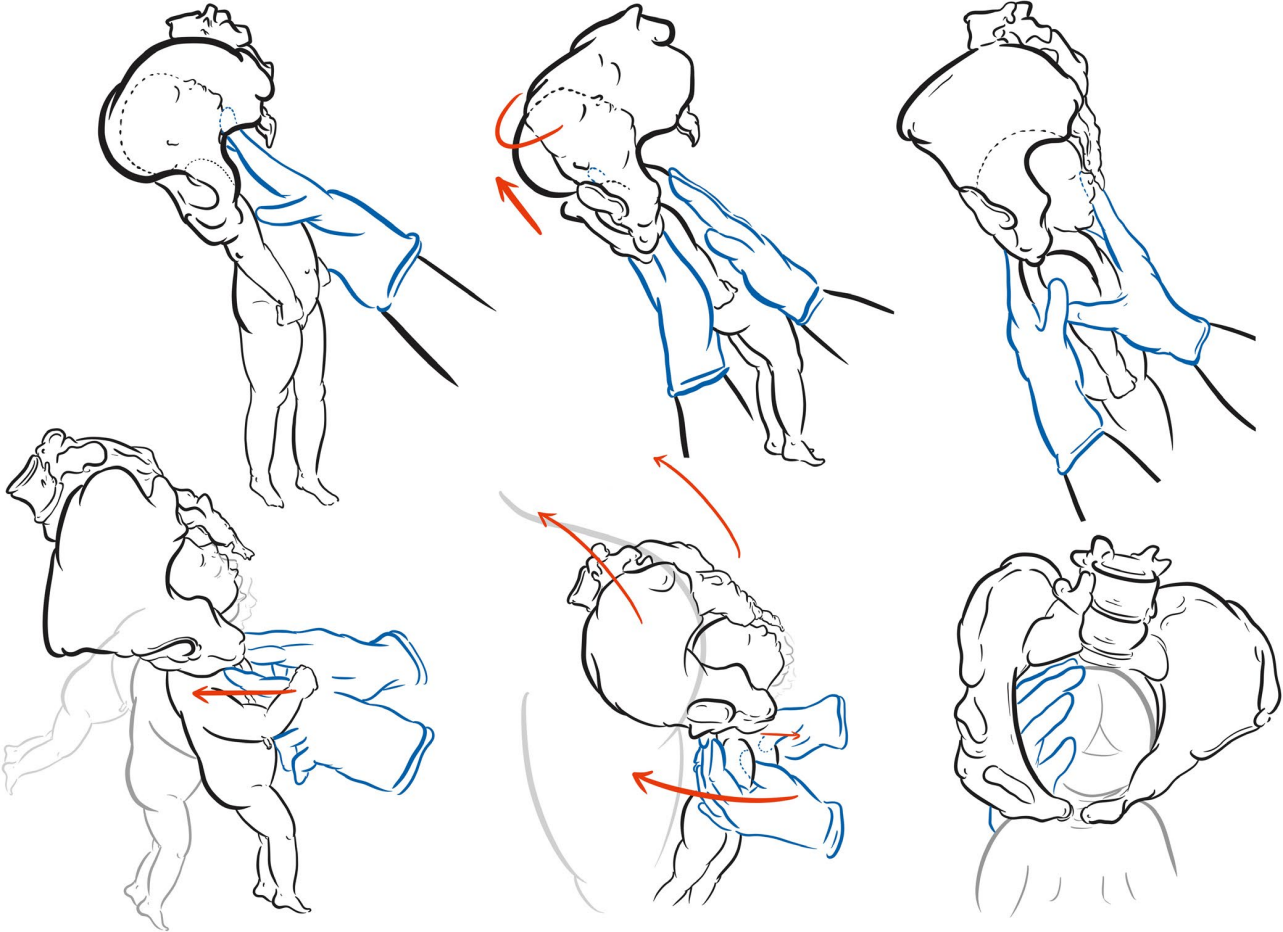
Assisting the birth of the head in physiological breech births. *Head at the pelvic inlet*: **Elevate and rotate** (top): (1) The birth attendant runs a finger up to identify that the chin is high; the head is extended and trapped at the inlet to the pelvis. (2) Using “flat hands” (also called “prayer hands”), the birth attendant shifts one hand onto the chest of the newborn. Another hand, on the back of the newborn, shifts up to elevate and lift the occiput off the maternal pubic bone. If necessary, the occiput would be rotated at this point into oblique or transverse to assist engagement. (3) Once engaged in the pelvis, the neonatal head is flexed and realigned in the pelvis. The head is then delivered by a shoulder press or variation of Mauriceau. *Head in the midpelvis or outlet *: **Shoulder press** (bottom) flexes the fetal head by moving the newborn's shoulder girdle and body toward the maternal abdomen, much like supine maneuvers to deliver the head. The pubic bone becomes a fulcrum, which lifts the occiput as the head pivots around the maternal sacral curve. This is performed either by pressing on the fetal chest, just below the clavicle, or with thumbs on the fetal chest and fingers wrapped around the shoulders. **Buttock lift** augments the effectiveness of shoulder press by slightly elevating the maternal sacrum, enlarging the anterior‐posterior diameter of the pelvic outlet, and sweeping the perineum over the newborn's forehead. **Scoop and flex** can be used if the above are not successful, or to align the head in the pelvis. The birth attendant sweeps one hand over the parietal bone and flexes the head down by pressing on the sinciput (forehead). *Drawings* by Merlin Strangeway, Drawn to Medicine

Our findings have been translated into an algorithm by Shawn Walker (Figure 2) containing an initial description of what we consider to be safe parameters for timekeeping and indications for interventions in physiological breech births. The algorithm was circulated via teaching exercises, revised, and translated following usability feedback from professionals using physiological breech birth in their practice within UK National Health Service and international hospitals, over an 18‐month period.^16^, ^19^ Although the algorithm was not tested as part of this study, evaluations in settings where it is used are currently underway. Maneuvres for relieving head entrapment at the pelvic inlet, mid‐pelvis and outlet are illustrated in Figure 3.

## 4 DISCUSSION

In historical obstetric practice, vaginal breech births have been managed with the woman in a supine position. However, as “standard” practice has declined in preference for delivery by cesarean birth, many centers that have re‐implemented vaginal breech births are discovering the benefits of upright maternal positioning.^2^, ^16^, ^19^, ^20^ This has led to modifications of traditional maneuvers, as observed in our study, that enable attendants to provide timely assistance when required.

Our close analysis of 42 upright breech births suggests that spontaneous breech births occur very quickly once the buttocks have descended past the ischial spines and onto the perineum. The data suggest that delay of 90 seconds or more at any point after “rumping” indicates a completely spontaneous birth is unlikely. In this sample, even most assisted births were completed within 3 minutes of the birth of the fetal pelvis. The 2017 Royal College of Obstetricians and Gynaecologists (RCOG) guideline suggests:

In general, intervention to expedite breech birth is required if there is evidence of poor fetal condition or if there is a delay of more than 5 minutes from delivery of the buttocks to the head, or of more than 3 minutes from the umbilicus to the head. (p.17) 2

We suggest that for upright breech births, a delay of 90 seconds or more occurring at *any* point during emergence indicates a completely spontaneous birth is unlikely and consideration should be given to assisting the birth. Encouraging maternal movement and effort *before* performing hands‐on interventions will help to spontaneously resolve minor delays, or to confirm the need for manual assistance.

Video analysis has been used by other research teams in related studies around the time of birth. Zhang et al^21^ recorded 92 videos of normal cephalic births with normal infant outcomes. They found that the average head‐to‐body delivery interval was 71.04 ± 61.02 seconds, suggesting the timings of cephalic and breech births may be similar.

The mechanisms of vaginal breech birth have been described by others.^4^, ^15^, ^17^, ^22^ Our observations concur with Plentyl and Stone^22^ and Louwen et al,^15^ in that the arms were most often born elbow‐first, folding across the midline of the body, with the shoulders in the transverse diameter in a majority of our videos. This differs from Evans’ description^4^ of the anterior arm being born under the symphysis pubis after a rotation; in our data set, this occurred more occasionally. We also observe that in most cases, the sacro‐anterior rotation of the fetal sacrum to the maternal pubis does not complete until the fetus has birthed to the line of the nipples/scapulae, rather than immediately after birth of the pelvis. We have also not observed that beginning with the back on one side or the other (left/right) is more optimal for a vaginal breech birth.^4^

This is the first paper to report timings and variations in fetal mechanisms during the final moments of upright breech births with this level of detail, which cannot be captured in even the most comprehensive contemporaneous notes. Previous studies of breech births in upright maternal positions have reported higher rates of completely spontaneous births than we have. Bogner et al^23^ reported 70.7% (29/46) of births in “all fours” occurred without interventions, and Louwen et al^15^ reported 56% (129/229). The reason for this discrepancy is unclear. Practitioners may not record all maneuvers accurately. For example, maneuvers that require minimal effort, for example, a very simple shoulder press, may not seem significant enough to record, similarly to routine axial traction in supine cephalic births.

Our work suggests that the strategy of instructing the woman to breathe through her urge to push while “waiting for the next contraction” should be reconsidered. When the fetus is delivered to the shoulders, the fetal head should be in the maternal pelvis or needs urgent assistance to engage. Because the uterine myometrium is no longer stretched, the positive feedback cycle that stimulates physiological labor progress^24^ begins to change, contractions space out, and the uterus initiates third stage, placental delivery.^25^ Uterine contractions stimulate the maternal urge to push, but the fundus itself is no longer the mechanism of fetal expulsion; maternal effort is key. Unnecessary delay may increase the chances that the placenta will detach from the uterine wall before the birth of the fetal head.

Experienced practitioners have suggested that when women birth in an upright, forward‐facing position, such as kneeling, it is easier to observe for signs that the delivery will be more difficult.^2^, ^4^, ^15^ Our study has confirmed what some of those signs may be, based on empirical evidence. Obstetricians and midwives learning breech birth skills for the first time may find learning theory with physiological methods useful.^26^, ^27^ Although obstetricians who have been in practice for many years may be more familiar with performing maneuvers with the woman in a dorsal position,^2^ our analysis indicates that conversion to lithotomy can be accomplished very quickly, confirming the safety of offering mobile women a choice of initial birthing position.^2^, ^28^, ^29^ However, only 10% of the women in our sample used epidural anesthetic, and this strategy may not work when women are affected by a heavy epidural block.

Whereas the fetal pelvis will usually emerge with the sacrum pointing in a transverse or anterior oblique direction, lack of rotation to a direct sacro‐anterior position by the time the fetus has emerged to the nipple line (when the scapulae would be apparent in a supine birth) was associated with the performance of rotational maneuvers to release an extended fetal arm. Physiological breech birth strategies involve purposeful noninterference if the birth is progressing normally,^11^ while assisted supine breech delivery training programs recommending manually “correcting” to sacro‐anterior after the birth of the fetal pelvis.^30^ In this data set, arms were only assisted by sweeping down in 12/42 (26%) of births, and only 6/42 (14%) involved rotational maneuvers. A similar study, to determine whether or not manually “correcting” the orientation of the fetal pelvis earlier in the birth results in a higher or lower incidence of assisting the birth of fetal arms, would be useful.

When confronted with a delay of fetal arms in the midpelvis, clinicians in this sample tended to attempt the release of the sacral arm first. This is possibly because of its proximity to the clinician in upright births, or a belief that reaching the arm will be easier with more space under the sacrum. We have begun to teach novices to attempt release of the pubic arm first, based on our observations and experience in practice. Further research should systematically explore whether releasing the pubic arm first is a more efficient and successful strategy for “restoring the mechanism.”^31^

We observed that clinicians expedited the birth of the fetal legs in 7/42 cases. Each time this was done by lifting the perineum or maternal buttocks, and in each case, subsequent interventions were performed to deliver the arms and/or head. After reviewing the videos, we suggest that if encouragement of maternal movement and effort is insufficient to release extended legs, gentle pressure on the popliteal fosse may release the legs more easily and may cause less disturbance for the woman.

In 5/42 (12%) cases, clinicians used a finger in the fetal mouth to flex the head. This strategy is often discouraged in the performance of the Mauriceau‐Smellie‐Veit/Mauriceau‐Cronk maneuver to avoid the theoretical risk of jaw dislocation or distocclusion, in which the lower jaw becomes positioned in a distal or posterior position in relation to the upper.^32^ However, because the mandible joins the skull at the base of the occiput, this does aid flexion by elevating the occiput. Like every maneuver, it should be used with caution, only after determining that the head is engaged, and outcomes should be monitored closely.

### 4.1 Strengths and limitations

Because we used videos to which we had historical access for teaching/research purposes but without linking personal demographics, we did not correlate our observations with parity or detailed fetal outcomes. According to those who provided them, all fetal outcomes were positive, even among the videos which contained complications. We did not exclude any videos we had permission to use. Very complicated births may have provided comparative insight into what is “normal for breech,” but their inclusion is unlikely to have affected our conclusions about what happens in the majority of spontaneous upright breech births. Additionally, our findings relate to upright breech births only, and not to breech births in other maternal positions. We advocate a larger, prospective study, including births in supine maternal positions, detailed outcome data, and careful ethical arrangements around informed consent.

The parameter for which we have the least evidence is the interval between “rumping” and the birth of the head. This is because most video footage in our sample begins after this point. At this point, cord occlusion is more likely^2^ and monitoring the fetal heart becomes more difficult by external cardiotocograph because of the positioning of the fetal heart behind the maternal pubic bone. In some videos, the cardiotocograph is audible even after the fetal chest has emerged, clearly recording maternal heart rate. Therefore, we have included the “rumping” parameter as one which warrants observation. We encourage documentation to clearly record when the fetal buttocks remain visible on the perineum between contractions. Where video recordings are made for research purposes, they should be started before this point.

### 4.2 Conclusions

The Physiological Breech Algorithm designed by Walker accounts for expected timings in a normally progressing breech birth, as well as time required to perform maneuvers in cases of obstruction. We suggest this can be used to guide further research and key clinical documentation points such as the time of “rumping” (descent to +3 station, fetal buttocks visible between contractions); birth of the fetal pelvis; birth of the umbilicus; and encouragement of maternal movement and effort to confirm obstruction before initiating “hands‐on” interventions. Future studies concerning the safety of vaginal breech birth should clarify whether this algorithm is used to guide timekeeping and intervention strategy, or if not, which alternative parameters have been used.
