# Supplementary material for: Practical insight into upright breech birth from birth videos: A structured analysis
Source: Birth. 2020 Jan 20;47(2):211–9. doi: 10.1111/birt.12480 (PMC7318698; doi:10.1111/birt.12480)
Supplement: Supplementary file 1 [file BIRT-47-211-s001.docx]

**Supplementary materials published online only:**

**Table.** Descriptive data for physiological breech birth video series

| **Category** | **Variable** | **Incidence / 42 (%)** | **Births with indirect manoeuvres** | **Births with direct manoeuvres** | **Total births with interventions** |
| --- | --- | --- | --- | --- | --- |
| Total Births | With interventions | * | 15 (36%) | 28 (67%) | 32 (76%) |
| Attending Professional | Obstetrician | 34 (81%) | 15 (44%) | 34 (71%) | 28 (82%) |
|  | Midwife | 8 (19%) | 0 | 4 (50%) | 4 (50%) |
| Types of breech presentation | Frank/extended | 29 (69%) | 11 (38%) | 19 (65%) | 21 (72%) |
|  | Flexed/complete | 4 (10%) | 1 (25%) | 3 (75%) | 3 (75%) |
|  | Incomplete/one leg extended | 2 (5%) | 1 (50%) | 1 (50%) | 2 (100%) |
|  | ‘Dropped leg’/one leg down | 6 (14%) | 2 (33%) | 4 (67%) | 5 (83%) |
|  | Uncertain | 1 (2%) | 0 | 1 (100%) | 1 (100%) |
| Birth Setting | Hospital  Home or uncertain | 35 (83%)  7 (17%) | **Direct manoeuvres:**  *For arm(s) trapped at pelvic inlet:*   - rotational manoeuvres to release an entrapped arm   *For arm(s) caught mid-pelvis:*   - sweeping down the arm/s   *For extended head at inlet:*   - elevate & rotate fetal head to assist engagement   *For head at outlet/mid-pelvis:*   - shoulder press - Mauriceau-Cronk/modified Mauriceau   **Indirect manoeuvres:**   - episiotomy - manually ‘stretching’ the perineum - fundal pressure - maternal buttock lift performed independently of shoulder press | | |
| Lighting | Bright or daylight  Dim | 29 (69%)  13 (31%) |  |  |  |
| Use of epidural pain relief | Yes – mobile epidural  No | 4 (10%)  38 (90%) |  |  |  |
| Monitoring | Cardiotocograph  Hand-held doppler  Uncertain | 32 (76%)  4 (10%)  6 (14%) |  |  |  |
| Maternal birthing positions | Kneeling  Hands/knees or ‘all fours’  Standing  Knees/elbows | 25 (60%)  13 (31%)  2 (5%)  2 (5%) |  |  |  |
| Symmetry of birthing position | Symmetrical  Asymmetrical – eg. one foot and one knee planted | 38 (90%)  4 (10%) |  |  |  |
| Maternal movement | Spontaneous / undirected  Directed by caregiver  None | 10 (24%)  28 (66%)  4 (10%) |  |  |  |

**Figure.** Histogram with normal distribution curve, showing times between birth of umbilicus and completed birth

**
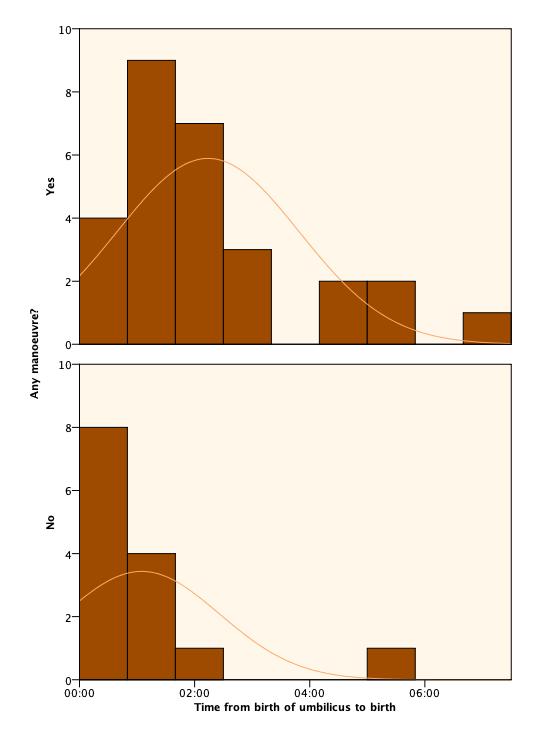
**

**Key Terms**

**Physiological breech birth:** A breech birth in which the woman is encouraged to remain upright and active throughout her labour and supported to assume the position of her choice for the birth. Gravity is an important natural force in alignment with the direction of birth.

**Upright breech birth:** A breech birth in which the woman births in an upright position, including: kneeling, hands/knees (all fours), standing, squatting, upright on a birthing stool, asymmetric (one knee/one foot planted), etc.

**Spontaneous birth:** In this study, ‘spontaneous birth’ refers to a birth in which no manipulation of the emerging infant occurred.

**‘Dropped leg’ presentation:** A variation of complete breech presentation, in which the fetus begins descent in a complete (two hips flexed) breech presentation but one leg extends downward during descent, prior to rumping. This commonly occurs around the time of full dilatation due to increased space under the sacrum. The fetal pelvis is engaged at this point, distinguishing a ‘dropped leg’ presentation from a true footling.

**Rumping:** The breech equivalent to ‘crowning,’ when the fetal buttocks have descended to +3 station and remain visible without receding between contractions. This is sometimes called ‘the point of no return,’ due to the understanding that this is the largest part in a normally grown term baby, and it has now passed the ischial spines.

**Sacro-anterior:** This refers to an orientation of the fetal sacrum towards the maternal pubis, regardless of maternal positioning. When women are upright, the sacrum will move away from the birth attendant, towards the maternal pubis.

**Pubic arm:** The fetal arm positioned closest to the maternal pubis.

**Sacral arm:** The fetal arm positioned closest to the maternal sacrum.

**Shoulder press:** A manoeuvre to deliver the aftercoming fetal head in an upright birthing position using pressure just below the clavicle to move the shoulder girdle back between the mother’s legs, flexing the fetal head.

**Buttock lift:** An indirect (without touching the fetus) manoeuvre to assist the delivery of the aftercoming head in an upright breech birth. It is performed by an assistant while the attending clinician performs shoulder press and involves lifting the mother’s buttocks up and away from the perineum. This lifts the sacrum to create space in the pelvis and sweeps the perineum over the forehead.

**Elevate and rotate:** A manoeuvre to resolve an extended fetal head that is trapped in direct OA or OP position at the pelvic inlet in a breech birth. It is performed by elevating the fetal head at the occiput to raise it off the pelvic inlet and/or internal manual rotation of the occiput to oblique/transverse to assist the head to engage, then rotating the head back to the OA diameter to realign in the mid-pelvis to deliver the fetal head.

**Scoop and flex**: Internal flexion of the fetal head by sweeping one hand over the parietal bone and pressing down on the sinciput (forehead).

**Flat hands**: The birth attendant places one flat hand on the anterior aspect of the fetal torso, and one on the back, with finger pads along the clavicle and shoulder girdle, in order to perform rotational manoeuvres to release entrapped arms.

**Shoulder girdle grip**: The birth attendant grips the fetal shoulder girdle with thumbs anterior and fingers wrapped around the shoulder blades, in order to perform rotational manoeuvres to release entrapped arms.
